# ScRNA-seq and spatial transcriptomics: exploring the occurrence and treatment of coronary-related diseases starting from development

**DOI:** 10.3389/fcvm.2023.1064949

**Published:** 2023-06-20

**Authors:** Can Liu, Fan Yang, Xin Su, Zhenpeng Zhang, Yanwei Xing

**Affiliations:** Guang'anmen Hospital, China Academy of Chinese Medical Sciences, Beijing, China

**Keywords:** scRNA-seq, spatial transcriptomics, coronary, molecular mechanism, inflammation, precision medicine

## Abstract

Single-cell RNA sequencing (scRNA-seq) is a new technology that can be used to explore molecular changes in complex cell clusters at the single-cell level. Single-cell spatial transcriptomic technology complements the cell-space location information lost during single-cell sequencing. Coronary artery disease is an important cardiovascular disease with high mortality rates. Many studies have explored the physiological development and pathological changes in coronary arteries from the perspective of single cells using single-cell spatial transcriptomic technology. This article reviews the molecular mechanisms underlying coronary artery development and diseases as revealed by scRNA-seq combined with spatial transcriptomic technology. Based on these mechanisms, we discuss the possible new treatments for coronary diseases.

## 1. Introduction

Coronary arteries are the only arteries that supply blood to the heart. Coronary artery disease may lead to insufficient blood and oxygen supply to the heart, resulting in myocardial necrosis, which can be fatal in severe cases (1). According to data on the official website of the World Health Organization, an estimated 18 million people died from cardiovascular diseases in 2019, representing 32% of all global deaths. Owing to the lack of understanding of the microscopic changes in coronary artery disease, the molecular mechanisms and gene expression levels during disease progression are at an exploratory stage. Identifying specific gene expression or targets in coronary lesions facilitates development of precision medicine (2, 3).

With the invention of single-cell transcriptomics, over the past decade, researchers have been able to explore coronary development and disease processes from a single-cell perspective. Single-cell RNA sequencing (scRNA-seq) is a powerful tool that can generate and validate many predictions that need to be confirmed experimentally (4–6). However, given the technical confounding factors and the disordered nature of sequencing, scRNA-seq data do not necessarily represent the ground truth regarding biological differences. Therefore, the accuracy of the results must be validated further. With innovations in single-cell sequencing technology, single-cell spatial transcriptomic technology that preserves the spatial information of single cells has also been constantly updated (7, 8). According to technical implementation, there are common technologies that combine RNA *in situ* hybridization or paired cell sequencing or complex algorithms with scRNA-seq data (9, 10). The latest technology is a direct single-cell spatial transcriptomic technique in which scRNA-seq is performed on living tissue sections using spatially encoded RNA capture probes. These technologies can be used to obtain the spatial information of a single cell of a certain organization. RNA *in situ* hybridization, including single-molecule fluorescence *in situ* hybridization (smFISH), complex computational algorithms, and cell pairing, is based on the principle that some components of spatial information are compared with scRNA-seq data to obtain the spatial information of a single cell (8, 11, 12). This study focused on single-cell spatial transcriptomic sequencing and RNA *in situ* hybridization combined with scRNA-seq sequencing technology. (Table 1) These technologies can be used to directly validate the results of single-cell sequencing (11, 19). Furthermore, coronary artery disease, especially coronary atherosclerotic lesions, can easily cause myocardial infarction, which causes the myocardial cells around the infarction to have different degrees of ischemia and hypoxia. Hence, single-cell spatial transcriptomic techniques can be used to identify the spatial differences in lesions around the infarct.

**Table 1:** Match single-cell spatial transcriptomics with references.

Methods	Required input data other than scRNA-seq	Developmental stage	Species	Refs.
In Situ Hybridization	RNAscope	Embryonic (6.5 PCW)	Mouse	(13)
	RNAscope	Embryonic day 10.5-postnatal day	Mouse	(14)
	RNAscope	Adult	Human	(15)
	RNAscope	Adult	Mouse	(16)
	RNAscope	Embryonic (E12.5-E17.5)	Mouse	(17)
10X Visium	None	Embryonic (E9.5-E12.5)	Mouse	(18)

In this study, we explored the findings of scRNA-seq and spatial transcriptomic techniques in a multicellular coronary microenvironment. We integrated recent results from single-cell sequencing and spatial transcriptomic studies of coronary developmental stages and diseased coronary arteries to provide the latest insights into the cellular origins and molecular mechanisms of coronary artery disease development and coronary lesions. We found that cells such as venous sinuses are the major contributors to coronary development during the developmental stages. Furthermore, epicardial cells play key roles in promoting the development of coronary arteries. Focusing on the composition of the pathological microenvironment in coronary atherosclerotic lesions, we discovered a much-debated collateral circulation and its formation. Various types of cardiac cells participate in a coronary-capable collateral circulation through different mechanisms. Based on the mechanism targets and gene expression discovered using single-cell spatial transcriptomics technology, we propose possible molecular targets and methods to achieve precision medicine for coronary artery disease, with the hope of providing prospective future precision medicine treatment ideas.

### 1.1. Using scRNA-seq to identify progenitor cells in the context of coronary development

Determining the mechanisms underlying coronary arterial origin has always been a striving direction for researchers (20, 21). With progress in research methodologies, researchers have deepened their understanding of the origin of coronary arteries.

**Figure 1:**
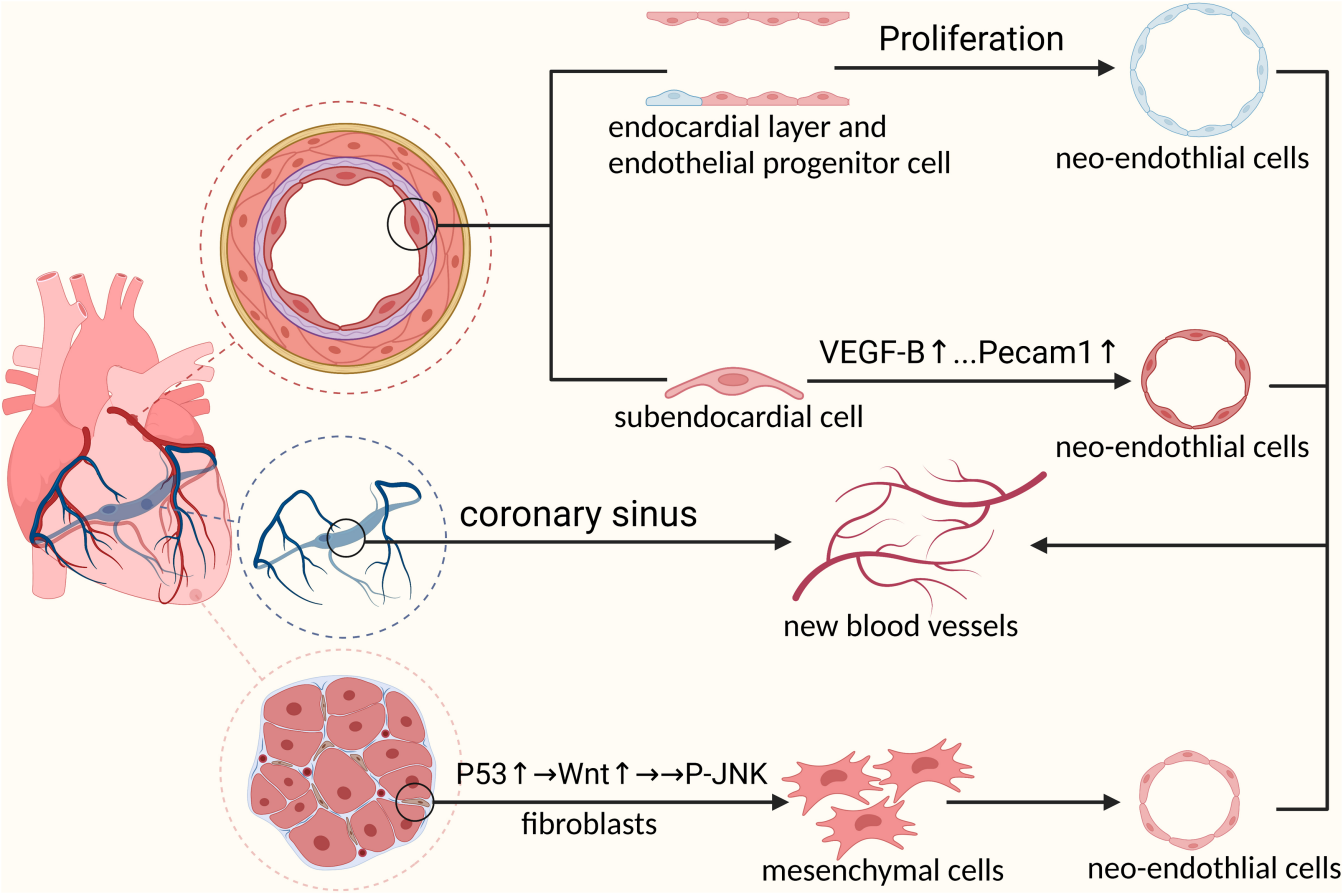
Mechanisms of embryonic coronary artery development: 1. Venous sinus cells must first differentiate into pre-arterial cells before participating in coronary artery formation 2. Prdm6 promotes cardiac neural crest cells to differentiate into pericytes through epithelial-mesenchymal transformation by upregulating the expression level of WNT1. Next, pericytes differentiate into coronary smooth muscle cells under the activation of NOTCH3 signal to participate in coronary artery formation 3. Endocardial cells involved in coronary formation during embryonic development.

From the earliest “aortic sprouting origin” theory to the belief that there was a transient structure “precordial” in the embryo, coronary arterial origin has been studied for decades. Using single-cell sequencing technology, we identified the venous sinus origin of the coronary arteries. The formation of coronary vasculature is a complicated and unintuitive process involving multiple cellular sources. Advances in single-cell sequencing technologies have allowed us to analyze the different origins of coronary arteries at the single-cell level. Studies have shown that the venous sinus, endocardium, and cardiac neural crest are the main sites of coronary artery development. In addition, epicardial cells express vascular chemokines that promote coronary artery development in a paracrine manner (Figure 1).

Studies have suggested that the venous inflow channels of the embryonic heart, that is the venous sinuses, migrate and differentiate into coronary arteries (22). To explore the mechanisms and specific markers of the sinus-to-coronary transition, researchers have sequenced embryonic mouse hearts using scRNA-seq tools. Based on the cell clustering of sequencing data, the continuous and discrete cell clusters were calculated to determine the regularity of the cardiac developmental transition. Finally, the researchers proposed the concept of “ pre-arterial population” and discovered that a new gene, *Slc45a4*, marked the pre-arterial population at an early stage (22). *Slc45a4* was enriched in adult coronary arteries. They suggested that embryonic sinus cells would leave the sinus and differentiate into coronary arteries by gradually reducing their venous identity and enhancing arterial gene expression. Venous sinus cells reach the gain threshold of arterial genes and form a cell group that resembles mature coronary arteries. This cell population exists before appearance of coronary blood flow and is no longer observed after the embryonic coronary arteries are formed. Therefore, the population is distinct from mature coronary plexus cells and is known as a pre-arterial population. On induction of the expression of COUP-TF2 to block the formation of anterior arterial populations, the veins fail to develop into arteries (22).

In addition to sinus venosus, studies have suggested a modest contribution from the ventricular and atrial endocardium. This shows that the process of pre-arterial grouping is indispensable during maturity and development of the coronary arteries (23). According to previous research, the postpartum cardiovascular system undergoes a process of trabecular compaction. During this process, neocoronary circulation occurs along with the rapid formation of the ventricle. The neocoronary vasculature has been proven to be largely *de novo* rather than derived from the original vasculature. Tian et al. used genetic lineage-tracing approaches to demonstrate that the endocardium plays an important role in neocoronary circulation (23). There are many discrepancies in the formation process, vascular morphology, and spatial location of blood vessels from different sources. These discrepancies would affect the physiological state and pathological processes in blood vessels. To investigate functional differences between vessels of different origins, scRNA-seq analysis could be a powerful tool to provide substantial clues in identifying these discrepancies (23).

Cardiac neural crest cells are a transient population of embryonic pluripotent cells unique to vertebrates. In recent decades, researchers have discussed the role of cardiac neural crest cells in heart development, including coronary artery development. Recent studies have shown that cardiac neural crest cells are directly involved in the formation of coronary muscle cells (24). Chen et al. performed lineage tracing using single-cell RNA transcription technology and found that pericytes derived from cardiac neural crest cells had the ability to gradually migrate and differentiate into vascular smooth muscles (14). Notch3 signaling is significantly upregulated during the migration of cardiac neural crest cell-derived pericytes to vascular smooth muscle cells (14). The Notch3 signaling ligand, Jagged-1, is induced following the onset of blood flow. Notch3 binds to Jagged-1 to guide pericoronary vascular smooth muscle cell differentiation (25). A scRNA-seq study found that cardiac neural crest cells undergo an epithelial-mesenchymal transition during embryonic development (26). During this process, neural crest cells migrate and differentiate into different cell types. Loss of the epigenetic modifier Prdm6 impairs the epithelial-mesenchymal transition of cardiac neural crest cells, resulting in the failure of normal heart development. Correlative changes have been observed in the expression levels of Prdm6 and WNT1 at different stages of cardiac neural spine cell migration and differentiation. Researchers have speculated that the mechanism by which Prdm6 affects heart development may be related to WNT1 signaling levels (26). Gandhi et al. used scRNA-seq to verify that the transcription factor Tgif1 is required for the formation of cardiac neural crest cell populations (27). In situ hybridization was used to measure the expression of Tgif1, and the important role of the transcriptional cascade among Tgif1, Sox8, and Ets1 in determining the cardiac neural crest identity was verified (26). Based on the extensive migration and differentiation capabilities of cardiac neural crest cells, scRNA-seq combined with spatial transcriptomics is expected to lead to profound discoveries regarding the developmental mechanisms of cardiac neural crest cells. This is important for improving cardiac developmental cognition and for developing new diagnostic and therapeutic approaches (18).

During cardiac development, the epicardium-derived cells express vascular maturation factors that promote coronary artery development. Simultaneously, a subset of the epicardium undergoes an epithelial-mesenchymal transition to give rise to a cell population that differentiates into multiple cell types, which we call epicardial-derived cells (28). Epicardial-derived cells undergoing epithelial-mesenchymal transition are involved in the maturation of coronary arteries. The process of epicardial epithelial-mesenchymal transition is integral to normal coronary development. Blockade of epicardial epithelial-mesenchymal transition results in incomplete and malformed coronary arteries. Jackson-Weaver et al. showed that an epicardium lacking protein arginine methyltransferase 1 (PRMT1) affects the formation of coronary arteries (29). Using scRNA-seq, they found that the lack of PRMT1 resulted in an abnormal increase in P53. P53 inhibits cell division, thereby blocking the epicardial epithelial-mesenchymal transition. Simultaneously, they observed incomplete perivascular coverage of coronary smooth muscle cells in mice lacking PRMT1 and loss of perivascular cells in the ventricular wall. This is related to proangiogenic maturation factors expressed by epicardium-derived cells (30, 31). Vascular maturation factors interact with adjacent coronary cells via a paracrine pathway of epicardium-derived cells. The normal role of angiogenic maturation factor might activate the expression of some genes in the recipient cells, leading to normal cell division, proliferation, and differentiation into mature coronary arteries. In addition, epicardium-derived cells are believed to have the ability to differentiate into smooth muscle cells and potentially endothelial cells (25, 32). The exact target or mechanism by which the epicardium affects the formation and maturation of the coronary arteries is still under investigation. As technology develops, the methodological realization of scRNA-seq combined with spatial transcriptomics can serve as a powerful hypothesis-generation tool. Using this technique, we can better understand the specific receptor cells and targets of pro-angiogenic maturation factors in the paracrine pathway, as well as the downstream mechanisms of coronary artery formation after receptor cells are activated. This will help us further unravel the secrets of adventitial cells in coronary development and identify useful targets in coronary disease (18).

Moreover, scRNA-seq technology plays an important role in identifying key signaling targets during coronary development and maturation, such as NOTCH signaling pathway-related targets. NOTCH signaling is widely active during early embryonic development and can be maintained at low expression levels during maturation (33). NOTCH signaling participates in cell fate selection based on the binding of ligand receptors and maintains physiological homeostasis (34). During coronary development, abnormal NOTCH signaling can lead to severe malformations. Using single-cell sequencing data of human embryonic hearts, researchers have found that decreased expression of NOTCH-targeted genes in the vascular endothelium contributes to malformed coronary arteries after excluding interfering cells in transition clusters. Interestingly, the occurrence of coronary malformations can be reduced by upregulating NOTCH-targeting ligands, which inversely proves the important role of NOTCH-targeted gene expression in the normal development of coronary arteries.

### 1.2. Gathering spatial information from single-cell data to study coronary artery dysplasia

Single-cell transcriptome sequencing has unique advantages for detecting the expression in single cells. Single-cell transcriptome sequencing has laid the groundwork for identifying signal changes in individual cells, who cell types were previously unknown, and cell subpopulations during coronary artery development. However, owing to the technical limitations of scRNA-seq, the spatial information of single cells cannot be obtained during the separation process, masking the information exchange between cells. However, with the advancement in technology, the spatial information of single cells can now be obtained during the isolation process (12, 18). Spatial transcriptomics can deepen our understanding of cell-cell interactions during coronary development, maturation, disease, and aging. At present, some achievements have been made in the application of spatial transcriptomics to coronary artery development, which provides a valuable reference for understanding coronary artery development to explore coronary physiology and pathology and develop therapeutics (35).

Asp et al. found that differences in gene expression were more pronounced between regions within the heart during early embryonic development than between different time points (13). They found that distinct cell clusters of fibroblasts have well-defined regions of localization during cardiac development and are involved in the development of different tissues. Based on the differences in marker genes, Asp et al. performed dimensionality reduction clustering on the obtained single-cell data and divided the identified fibroblast-like cells into four types of cell clusters. Dissecting the spatial information of the four cell clusters of fibroblast-like cells revealed that each cluster was located at a different spatial location in the heart and was involved in different tissue development. One type of cell cluster, located in the atrioventricular subepicardial mesenchyme, participated in the formation of coronary arteries (23). Two other types of cell clusters were located in the subepicardial and outflow tracts and were involved in angiogenesis and arterial morphogenesis. In addition to fibroblasts, we performed spatial information analysis of two cell clusters of endothelial cells (13). One of these cell clusters was localized in the trabecular myocardium and was a component of small blood vessels. The second was located in the dense myocardium and was a component of the coronary arteries.

Cardiac neural crest cells can participate in the formation of coronary vascular smooth muscles. However, specific cardiac neural crest cell types and locations remain unknown. Chen et al. used single-molecule fluorescence *in situ* hybridization to identify the spatial distribution of different subsets of cardiac neural crest cell-derived muscle cells (14). One type of vascular smooth muscle cell cluster, C1, which expresses high contractility markers, was found to be located in the coronary arteries, whereas the other two cell clusters, C2 and C3, were located in the aorta. In addition, the levels of pericyte markers, such as G protein-regulated signaling protein 5 (Rgs5) and Kcnj8, were significantly higher in C1 cell clusters. This validates the findings from previous single-cell studies showing that cardiac neural crest cell-derived pericytes can differentiate into coronary vascular smooth muscle (14).

Thymosin *β*4 encoded by TMSB4X is a well-recognized pleiotropic secreted peptide that plays an important role in cytoskeleton formation and motility (36). To study the expression of thymosin *β*4 during cardiac development, researchers selected chicken hearts at different stages according to the developmental stages of the Hamburg-Hamilton ventricle for single-cell transcriptomic analysis. Mantri et al. performed cell type annotation based on single-cell clustering and found that thymosin *β*4 was ubiquitously expressed in all cell types (37). The expression levels of TMSB4X vary in different cell populations as development progresses. Single-molecule fluorescence *in situ* hybridization exploration revealed that differences in thymosin *β*4 levels were related to spatial organization (37). During early development, TMSB4X is enriched in the endocardium and epicardium, while thymosin *β*4 has the highest expression in the coronary vascular endothelium at the late developmental stages. As previously described, the epicardium promotes coronary development, and the coronary arteries originate from the endocardium. Based on this, we believe that TMSB4X is always enriched during coronary development and both before and after coronary formation. In other words, the high expression of thymosin *β*4 contributes to the formation and development of coronary arteries. Existing studies have found that increasing the content of thymosin *β*4 in mice after myocardial infarction can prevent cardiac damage and improve cardiac function (38). This may be related to the promotion of coronary regeneration by thymosin *β*4 (39–41). E xploring the relationship between TMSB4X, which is enriched during coronary artery development, and the regenerative capacity of the coronary artery will have pioneering significance in the treatment of coronary artery diseases, such as myocardial infarction.

We predicted that spatial transcriptomics might be useful for understanding epicardial paracrine mechanisms. Single-molecule *in situ* hybridization spatiotemporal analysis based on transcriptional data by Lupu et al. confirmed our hypothesis (42). Lupu et al. found that Rgs5-expressing epicardial-derived cells were tightly attached to coronary endothelial cells (42). Under this spatial structure, the PDGF-B signaling pathway in coronary endothelial cells is activated in a paracrine manner to induce the maturation of coronary wall cells. More abundant and mature single-cell spatial transcriptome studies may play a greater role in understanding epicardial paracrine mechanisms.

## 2. New insights into coronary artery disease based on scRNA-seq

Coronary artery disease, particularly coronary atherosclerosis, is characterized by high morbidity and mortality rates. Single-cell transcriptome sequencing studies have focused on disease progression and pathological changes in coronary atherosclerosis. scRNA-seq studies have demonstrated changes in endothelial cells, vascular smooth muscle cells, and immune cells during the pathological process of coronary artery diseases and systematically explored the sensitivity and plasticity of these cells to changes in the microenvironmen (2, 43).

According to previous studies, adult mouse arterial endothelial cells are primarily composed of three cell populations with different characteristics. These include mature vascular endothelial cells, endothelial cells with inflammatory properties, and endothelial cells with mesenchymal properties (44). Different types of endothelial cells are differentially transformed and play different roles in the development and prognosis of various diseases. Single-cell sequencing data suggest that endothelial cells are activated during coronary artery disease. Endothelial cells acquire different phenotypes under the stimulation of a pathological microenvironment. Together with immune cells, endothelial cells constitute an arterial inflammatory environment and promote atherosclerosis (45).

Furthermore, single-cell transcriptome studies have explored the changes in vascular smooth muscle during atherosclerotic pathology. A study found that in atherosclerotic lesions, vascular smooth muscle cells, which are contractile in the normal state, undergo dedifferentiation and result in differentially expressed cell clusters (46). These diverse cell types are involved in the formation of the complex environment of diseased blood vessels. In addition, scRNA-seq studies also confirmed that the content of Krüppel-like factor 4 (KLF4) and the expression level of the *Tcf21* gene in mice play crucial roles in the dedifferentiation of vascular smooth muscle cells into various cell types (47–49). Various derivatives of muscle cells have different functions that influence the disease process in a positive or negative manner (50). Continued exploration at the molecular level will help us understand the specific roles of different cell types in coronary atherosclerosis, which could contribute to development of precision medicine.

Inflammation is directly involved in the overall development of coronary atherosclerosis (51). In the early stages of the disease, a large number of immune cells are recruited to the diseased areas (52). Single-cell transcriptome studies have shown that different immune cell types exhibit functional differences in pathological changes, demonstrating immune cell diversity in lesions. Based on the clustering of single-cell sequencing data, different clusters of macrophages participate in different stages of inflammation by expressing different functional genes. Macrophages expressing inflammatory response-related factors such as CYBA, LYZ, and S100A9/8 are directly involved in the inflammatory response (53). The IFNIC macrophage population that prominently expresses Isg15, Irf7, Ifit1, and Ifit3 is characterized by type I interferon production (53). Macrophage clusters with upregulated JUNB and NFKBIA are involved in pro-inflammatory responses. Macrophage clusters are also involved in cholesterol uptake and lipid accumulation (54). T cells are present throughout the development of atherosclerotic lesions. The number of naïve CD4+ T cells decreases significantly during the lesion process, and they might differentiate into other types of T cells. CD8+ T cells show functional polarization during the pathological process, which may contribute to the pathological process and may also be vasoprotective. In addition to the inflammatory response of cells, scRNA-seq studies have shown that innate lymphocyte-2 has a strong vascular protective effect against atherosclerosis (52). Single-cell transcriptome sequencing has further revealed the phenotypic transformation of various cells in the pathological microenvironment of coronary atherosclerosis. Complex interactions between cells affect the progression of coronary artery disease and participate in the generation of the pathological microenvironment.

### 2.1. The latest view on coronary collateral circulation

After coronary artery disease occurs, insufficient blood supply to the heart makes the heart prone to myocardial infarction and cardiac dysfunction. Currently, revascularization therapy is the first-line treatment for coronary artery disease. However, this method of clinical revascularization is not suitable for all patients and can be applied in limited situations (55). Moreover, ischemia-reperfusion injury may occur after reperfusion therapy, which further damages the myocardial function. Historical data have documented the absence of myocardial infarction in some patients with chronic total coronary occlusion, suggesting the possibility of additional collateral circulation in coronary occlusion (56). Recent studies have confirmed that collateral circulation in cases of coronary occlusion could help the heart supply blood and reduce myocardial damage (57, 58). However, imaging-based studies have been limited to exploring the targets and signals that regulate collateral angiogenesis. Therefore, researchers have selected single-cell transcriptomics, which is advantageous for exploring molecular targets.

**Figure 2:**
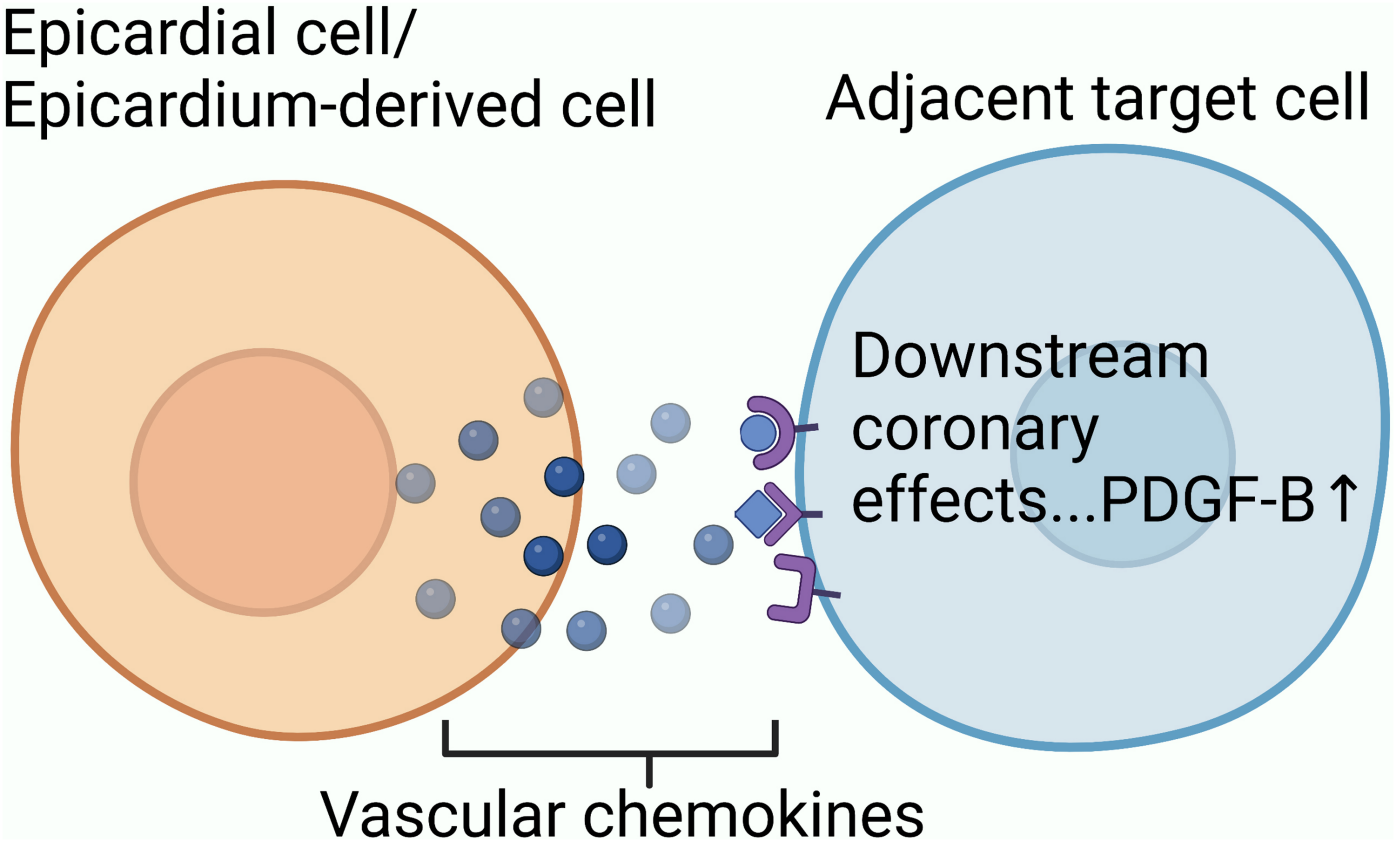
The mechanism underlying collateral circulation after myocardial infarction: endothelial progenitor cells, which still have differentiation potential in adult hearts, participate in initiating collateral circulation through proliferation and differentiation; P53-Wnt4-P-JNK mechanism promotes the epithelial-mesenchymal transformation of cardiac fibroblasts to form mesenchymal cells with differentiation potential. Mesenchymal cells proliferate and differentiate into arterial endothelial cells to participate in initiation of collateral circulation; Mature venous sinus cells and subendocardial cells after myocardial infarction also have the potential to differentiate into coronary cells.

Exploring the source of neovascularization after myocardial infarction might help us better understand collateral circulation. Based on single-cell transcriptome sequencing data, researchers have identified sources of neovascularization and related regulatory targets after myocardial infarction (Figure 2).

Endothelial progenitor cells, which retain their differentiation potential in the adult heart, are one of the main sources of neovascularization after myocardial infarction (59). Li et al. selected isolated mouse hearts for single-cell sequencing and found that endothelial progenitor cells in coronary resident endothelial cells after myocardial infarction could form new blood vessels via clonal proliferation (5). In this study, Li et al. discovered a novel endothelium-specific marker for marking new blood vessels, namely plasma vacuolar-associated protein (PLVAP). Suppression of PLVAP expression significantly inhibited the proliferation of new blood vessels. This suggests that the regulation of PLVAP expression could affect the formation of new blood vessels, providing a new potential target for therapy. Experiments by Ubil et al. in adult mice demonstrated that cardiac fibroblasts can re-experience a developmental transition, in which the heart undergoes an ischemic injury. It promotes neovascularization through the fibroblast-mesenchymal transition-endothelial process (60). Given the activity of the p53 transcription factor in various cellular stresses, researchers have explored whether p53 is involved in phenotypic changes in fibroblasts under myocardial infarction stress. They demonstrated that the transcription factor P53 could participate in the regulation of fibroblast mesenchymal-endothelial transition by introducing P53-binding primers (61). Dong et al. used chromatin immunoprecipitation and gene knockout methods to demonstrate that Wnt4 plays an important role in fibroblast transformation after cardiac injury. They found that Wnt4 was a key downstream target gene in the regulation of fibroblast mesenchymal-endothelial transition by the transcription factor P53. Under the action of the transcription factor P53, Wnt4 is overexpressed in cardiac fibroblasts and induces fibroblast mesenchymal-endothelial transition through the phosphorylation-JNK/JNK signaling pathway (61). Although some researchers thought that the method of labeling fibroblasts in their study was inaccurate, later investigators demonstrated that at least some fibroblasts undergo this transition (62). Compared with primer binding or gene knockout experiments, single-cell transcriptome sequencing can help us obtain relevant target signals more efficiently and accurately. Furthermore, single-cell transcriptome sequencing can be used to identify previously unidentified genes and reveal random allele expression. In a single-cell study, a population of cells co-expressing fibroblasts and endothelial cells was found in the post-MI cell population, suggesting that the transition from fibroblasts to endothelial cells precedes the mesenchymal transition. Currently, there is a lack of research on single-cell molecular mechanisms involved in the transition process. Dube et al. suggested that the coronary sinus plays an important role in *de novo* neovascularization after myocardial infarction (63). Considering that the coronary sinus is an adult derivative of the embryonic sinus, the activation of the adult coronary sinus to form new blood vessels after myocardial infarction may be related to the activation of embryonic gene expression. More in-depth single-cell transcriptome studies can help identify relevant targets. Räsänen et al. suggested that subendocardial endothelial cells participate in angiogenesis (64). In their study, they ligated mouse coronary arteries to simulate ischemic perfusion of mice hearts (65). Using tissue section staining and angiography, they demonstrated that high expression of the vascular endothelial growth factor-B (*VEGF-B*) gene promotes the proliferation of subendocardial endothelial cells to generate endocardium-derived coronary arteries (64). The same conclusion was reached in a lineage-tracing study. The researchers used scRNA-seq to analyze the mechanism by which VEGF-B regulates the proliferation and differentiation of mature cardiac endothelial cells into coronary vascular endothelium. They observed an increase in the capillary endothelium during proliferation (65). This suggests that the endocardium may undergo a transition to capillary endothelium during its proliferative transition to coronary endothelial cells promoted by VEGF-B. They also found increased expression of endocardial Pecam1, which is normally highly expressed in coronary endothelial cells (65). This suggests that under the expression of VEGF-B, the endocardium of the adult heart gradually overexpresses coronary genes. In addition, the expression of the capillary endothelial chemokine CXCL12 increases after myocardial infarction. The chemokine CXCL12, combined with the specific receptor CXCR4 located on the arterial surface, promotes the formation of new blood vessels.

### 2.2. Spatial information of epicardium and neovascularization

**Figure 3:**
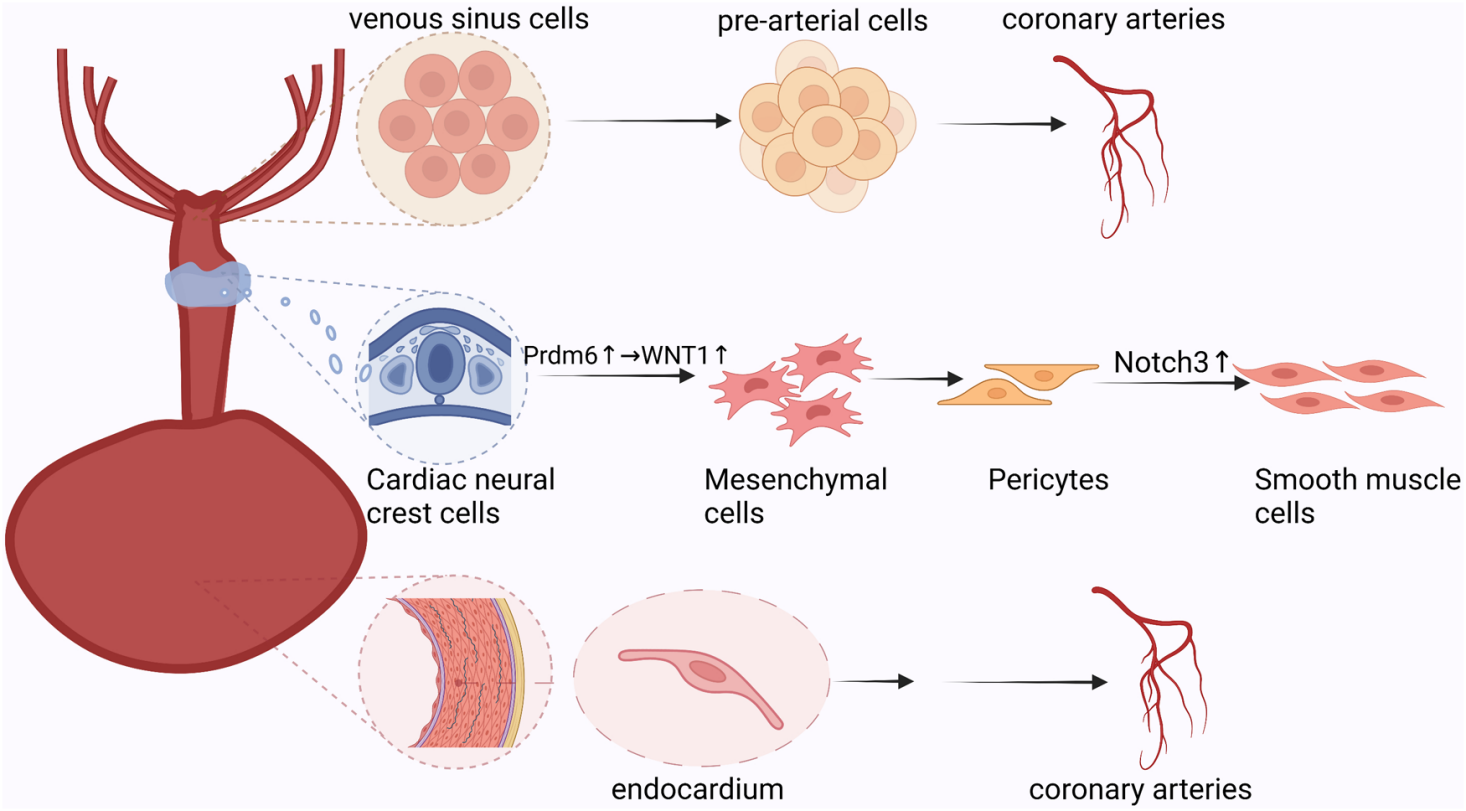
Paracrine process of epicardial cells: epicardial-derived mesothelial and mesenchymal cell populations secrete vascular chemokines that bind spatially adjacent downstream target cell receptors to promote neovascularization.

This study revealed the unique ecology of the border region surrounding the injured myocardium following myocardial infarction. There was a clear demarcation between injured and uninjured cells. Furthermore, there were spatial differences in the oxygen supply and demand balance of cardiomyocytes. Epicardial cells undergo multi-layered divisions in different hypoxic environments and participate in the formation of new blood vessels following myocardial infarction. Furthermore, the epicardium promotes neovascularization via the paracrine pathway by contacting spatially adjacent receptor cells (Figure 3) (15).

In a single-cell spatial transcriptomic study, Hesse et al. simulated myocardial infarction by ligating the left anterior descending artery in mice (16). All cardiac stromal cells were annotated using single-cell sequencing. Simultaneously, RNA *in situ* hybridization was used to display the spatial information of the different cell populations. They identified three populations of epicardial cells with distinct spatial preferences. Only clusters of epicardial cells expressing Sfrp2, Dkk3, and Pcsk6 were uniformly dispersed in infarct border areas. This confirmed that epicardial cells undergo multi-layered divisions under ischemia and hypoxia, which might be related to the different roles of different epicardial cell clusters in myocardial infarction. Deciphering the spatial distribution of the epicardium helps explain disease development and provides clues for treatment. Studies have shown that adult epicardial cells exposed to chronic hypoxia have developmental characteristics similar to the endothelial-mesenchymal transformation at the embryonic stage. They can differentiate into various cell types and promote coronary angiogenesis in a paracrine manner (66–68). To explore the role of endothelial-mesenchymal transition in the epicardium after myocardial infarction in angiogenesis, single-cell sequencing and RNA multiplex fluorescence *in situ* hybridization were combined (17). The relationship between epicardial cells and neovascularization has been discussed both cellularly and spatially. Researchers found that epicardial-derived mesothelial and mesenchymal cell populations overexpressed the vascular chemokines Sema3d and Slit2. The expression of these key vascular chemokines gradually increases during the development of epicardium-derived cells (17). Genes expressing different vascular chemokines guided differentiation of epicardium-derived cells into distinct cell populations and were localized and expressed in specific spaces. Researchers believe that the genes encoding vascular chemokines limit the developmental propensity of epicardium-derived cells. By blocking the epicardial endothelial-mesenchymal transition they found that Sema3d and Slit2 are reduced in epicardium-derived cells (17). Previous studies have demonstrated that the blockade of the epicardial endothelial-mesenchymal transition results in incomplete coronary and vascular endothelial development. Studies have confirmed that vascular chemokines and epicardial epithelial-mesenchymal transition promote mutually reinforcing relationships. Furthermore, vascular chemokines and epicardial epithelial-mesenchymal transition mutually promote and restrict each other, thereby promoting angiogenesis. Although the roles of epicardial epithelial-mesenchymal transition and vascular chemokines in coronary angiogenesis have been demonstrated, the ligand-receptor correspondence between vascular chemokines in the paracrine pathway and their downstream targets is not yet known. By constructing a receptor-ligand visualization, Quijada et al. found that the Robo4 receptor-expressing coronary endothelial cell population was mainly located in the Slit2-expressing epicardial mesenchymal cell population (17). Mesothelial cells expressing Sema3d are located on the surface of the heart (17). Epicardium-derived cells can affect coronary angiogenesis through receptor-ligand signaling. This study suggests that the differences of vascular chemokines highlight a complex link between epicardium-derived cells and coronary angiogenesis. Further research on the paracrine cues between changes in the epicardium and new blood vessels after myocardial infarction will provide new ideas for reperfusion therapy after myocardial infarction.

## 3. Spatial transcriptomics offers clues to therapy for damaged myocardium

Based on the current efforts in single-cell research on coronary artery development and disease, we propose the following new ideas for the treatment of coronary artery disease: 1. Targeting vascular chemokines 2. Induction of mature cardiac cells to reverse differentiation and proliferate to form new blood vessels 3. Induction of myocardial regeneration.

Human induced pluripotent stem cell therapy has shown great potential in experiments and has even been clinically applied (69). Single-cell information has been used to identify the subtypes of human induced pluripotent stem cell-derived endothelial cells to improve the efficiency of stem cell endothelial differentiation (70).

As an emerging molecular technology, spatial transcriptomics plays an important role in treatment of coronary artery disease. The coronary sinus, an adult derivative of the embryonic sinus vein, possesses vascularization ability similar to that of the sinus vein. Furthermore, adult coronary veins and arteries have the same origin during development (71). Based on these arguments, some researchers have hypothesized that adult coronary veins can transform into coronary arteries, and this hypothesis was tested using single-cell spatial transcriptomics. Some gene expression levels are related to cytogenetic changes, especially those of MECOM genes. McCracken et al. found that MECOM maintains coronary identity and argued that increased MECOM expression could drive the transition from veins to arteries. Research on MECOM may provide an effective therapeutic strategy for targeting and promoting angiogenesis (21). Based on the unique spatial properties of paracrine receptor-ligand binding, the study of epicardium regulating coronary artery formation through the paracrine pathway requires the aid of spatial transcriptomic techniques (72). Existing spatial transcriptome studies have confirmed that thymosin *β*4 plays an important role in promoting coronary vascular development. In order to explore a new method for the treatment of myocardial infarction, Chen et al. used an engineered exosome to continuously and slowly release thymosin *β*4 in the myocardial infarction area of model mice (73). It was found that thymosin *β*4 exosomes could significantly increase the level of neovascularization and restore blood supply after myocardial infarction (73). Echocardiographic comparisons also led to an analogous conclusion regarding improved cardiac function (74). A recent study proposed the concept of a cardiac progenitor cell bank to expand the lineage of cells capable of differentiating into cardiomyocytes for cardiac regenerative therapy following myocardial infarction. It is well known that immune inflammatory changes are involved in the coronary artery disease process. Single-cell studies have revealed that subsets of immune cells function differently during the disease course. Immune cells that express inflammatory factors participate in the inflammatory pathological environment, thereby exacerbating disease progression. There are, however, some distinct cell populations, such as a subset of CD8 T cells, that exhibit vasoprotective properties (52, 75). The identification of high number of different immune cells in single-cell omics studies helps to distinguish immune cell populations with cardioprotective effects during coronary artery disease, so as to seek new treatment methods.

## 4. Outlook and conclusion

Single-cell transcriptome sequencing is a revolutionary technology that has been successfully applied to different organs and tissues of an organism from an unprecedented perspective. Direct spatial transcriptome sequencing compensates for the loss of spatial information in single-cell sequencing and is not only used to verify the results of single-cell sequencing but also makes it possible to study the complex changes of coronary artery lesions in space. The biology of coronary artery disease involves multiple cell types and complex cell-to-cell interactions, and scRNA-seq allows for the detailed study of these multicellular microenvironments. scRNA-seq revealed important molecular mechanisms underlying the origin of coronary artery development and disease progression. Venous sinus cells must differentiate into pre-arterial cells before participating in coronary artery formation. Prdm6 promotes differentiation of cardiac neural crest cells into pericytes through epithelial-mesenchymal transformation by up regulating the expression level of WNT1. Next, pericytes differentiate into coronary smooth muscle cells under the activation of the NOTCH3 signaling pathway to participate in coronary artery formation. Furthermore, endocardial cells are also involved in coronary artery formation during embryonic development. However, current research on coronary artery disease is still identifying changes in gene expression levels in various cells at different stages, and research on various molecular mechanisms is still immature. Therefore, there is insufficient evidence regarding the accuracy of each molecular mechanism in disease progression. In the future, extensive spatial transcriptomic studies should be conducted to compensate for the lack of current molecular mechanistic studies (76). Based on the current findings, single-cell spatial transcriptomic techniques can be uniquely used to investigate the spatially multi-layered compartmentalization of epicardial cells that arises after coronary lesions. It is meaningful to use more spatial transcriptomic technologies to track transcription differences in infarcted hearts in the future. The spatial distribution obtained using this method is helpful for understanding the changes in gene expression related to cardiac remodeling after infarction. However, the challenge is to leverage this new knowledge and translate it into effective treatments to address the major clinical limitations in coronary disease.

## References

[1] Van Belle E Rioufol G Pouillot C Cuisset T Bougrini K Teiger E Investigators of the registre français de la FFR–R3F. Outcome impact of coronary revascularization strategy reclassification with fractional flow reserve at time of diagnostic angiography: insights from a large French multicenter fractional flow reserve registry. Circulation. (2014) 129(2):173–85. 10.1161/CIRCULATIONAHA.113.006646 24255062

[2] Ma WF Hodonsky CJ Turner AW Wong D Song Y Mosquera JV Enhanced single-cell RNA-seq workflow reveals coronary artery disease cellular cross-talk and candidate drug targets. Atherosclerosis. (2022) 340:12–22. 10.1016/j.atherosclerosis.2021.11.025 34871816 PMC8919504

[3] Jagadeesh KA Dey KK Montoro DT Mohan R Gazal S Engreitz JM Identifying disease-critical cell types and cellular processes by integrating single-cell RNA-Sequencing and human genetics. Nat Genet. (2022) 54(10):1479–92. 10.1038/s41588-022-01187-9 36175791 PMC9910198

[4] Lescroart F Wang X Lin X Swedlund B Gargouri S Sànchez-Dànes A Defining the earliest step of cardiovascular lineage segregation by single-cell RNA-Seq. Science. (2018) 359(6380):1177–81. 10.1126/science.aao4174 29371425 PMC6556615

[5] Li Z Solomonidis EG Meloni M Taylor RS Duffin R Dobie R Single-cell transcriptome analyses reveal novel targets modulating cardiac neovascularization by resident endothelial cells following myocardial infarction. Eur Heart J. (2019) 40(30):2507–20. 10.1093/eurheartj/ehz305 31162546 PMC6685329

[6] Suryawanshi H Clancy R Morozov P Halushka MK Buyon JP Tuschl T. Cell atlas of the foetal human heart and implications for autoimmune-mediated congenital heart block. Cardiovasc Res. (2020) 116(8):1446–57. 10.1093/cvr/cvz257 31589297 PMC7314636

[7] Williams CG Lee HJ Asatsuma T Vento-Tormo R Haque A. An introduction to spatial transcriptomics for biomedical research. Genome Med. (2022) 14(1):68. 10.1186/s13073-022-01075-1 35761361 PMC9238181

[8] Marx V. Method of the year: spatially resolved transcriptomics. Nat Methods. (2021) 18(1):9–14. 10.1038/s41592-020-01033-y Erratum in: Nat Methods. 2021 Feb;18(2):219 33408395

[9] Ren H Walker BL Cang Z Nie Q. Identifying multicellular spatiotemporal organization of cells with SpaceFlow. Nat Commun. (2022) 13(1):4076. 10.1038/s41467-022-31739-w 35835774 PMC9283532

[10] Rao A Barkley D França GS Yanai I. Exploring tissue architecture using spatial transcriptomics. Nature. (2021) 596(7871):211–20. 10.1038/s41586-021-03634-9 34381231 PMC8475179

[11] Moses L Pachter L. Museum of spatial transcriptomics. Nat Methods. (2022) 19(5):534–46. 10.1038/s41592-022-01409-2 35273392

[12] Li YH Cao Y Liu F Zhao Q Adi D Huo Q Visualization and analysis of gene expression in Stanford type A aortic dissection tissue section by spatial transcriptomics. Front Genet. (2021) 12:698124. 10.3389/fgene.2021.698124 34262602 PMC8275070

[13] Asp M Giacomello S Larsson L Wu C Fürth D Qian X A spatiotemporal organ-wide gene expression and cell atlas of the developing human heart. Cell. (2019) 179(7):1647–60. e19. 10.1016/j.cell.2019.11.025 31835037

[14] Chen W Liu X Li W Shen H Zeng Z Yin K Single-cell transcriptomic landscape of cardiac neural crest cell derivatives during development. EMBO Rep. (2021) 22(11):e52389. 10.15252/embr.202152389 34569705 PMC8567227

[15] Kuppe C Ramirez Flores RO Li Z Hayat S Levinson RT Liao X Spatial multi-omic map of human myocardial infarction. Nature. (2022) 608(7924):766–77. 10.1038/s41586-022-05060-x 35948637 PMC9364862

[16] Hesse J Owenier C Lautwein T Zalfen R Weber JF Ding Z Single-cell transcriptomics defines heterogeneity of epicardial cells and fibroblasts within the infarcted murine heart. Elife. (2021) 10:e65921. 10.7554/eLife.65921 34152268 PMC8216715

[17] Quijada P Trembley MA Misra A Myers JA Baker CD Pérez-Hernández M Coordination of endothelial cell positioning and fate specification by the epicardium. Nat Commun. (2021) 12(1):4155. 10.1038/s41467-021-24414-z 34230480 PMC8260743

[18] Saviano A Henderson NC Baumert TF. Single-cell genomics and spatial transcriptomics: discovery of novel cell states and cellular interactions in liver physiology and disease biology. J Hepatol. (2020) 73(5):1219–30. 10.1016/j.jhep.2020.06.004 32534107 PMC7116221

[19] Boogerd CJ Lacraz GPA Vértesy Á van Kampen SJ Perini I de Ruiter H Spatial transcriptomics unveils ZBTB11 as a regulator of cardiomyocyte degeneration in arrhythmogenic cardiomyopathy. Cardiovasc Res. (2022):cvac072. 10.1093/cvr/cvac072. [Epub ahead of print] PMC10064846 35576477

[20] Red-Horse K Ueno H Weissman IL Krasnow MA. Coronary arteries form by developmental reprogramming of venous cells. Nature. (2010) 464(7288):549–53. 10.1038/nature08873 20336138 PMC2924433

[21] Tian X Hu T Zhang H He L Huang X Liu Q Subepicardial endothelial cells invade the embryonic ventricle wall to form coronary arteries. Cell Res. (2013) 23(9):1075–90. 10.1038/cr.2013.83 23797856 PMC3760626

[22] Su T Stanley G Sinha R D'Amato G Das S Rhee S Single-cell analysis of early progenitor cells that build coronary arteries. Nature. (2018) 559(7714):356–62. 10.1038/s41586-018-0288-7 29973725 PMC6053322

[23] Tian X Hu T Zhang H He L Huang X Liu Q Vessel formation. De novo formation of a distinct coronary vascular population in neonatal heart. Science. (2014) 345(6192):90–4. 10.1126/science.1251487 24994653 PMC4275002

[24] Arima Y Miyagawa-Tomita S Maeda K Asai R Seya D Minoux M Preotic neural crest cells contribute to coronary artery smooth muscle involving endothelin signalling. Nat Commun. (2012) 3:1267. 10.1038/ncomms2258 23232397

[25] Volz KS Jacobs AH Chen HI Poduri A McKay AS Riordan DP Pericytes are progenitors for coronary artery smooth muscle. Elife. (2015) 4:e10036. 10.7554/eLife.10036 26479710 PMC4728130

[26] Hong L Li N Gasque V Mehta S Ye L Wu Y Prdm6 controls heart development by regulating neural crest cell differentiation and migration. JCI Insight. (2022) 7(4):e156046. 10.1172/jci.insight.156046 35108221 PMC8876496

[27] Gandhi S Ezin M Bronner ME. Reprogramming axial level identity to rescue neural-crest-related congenital heart defects. Dev Cell. (2020) 53(3):300–15. e4. 10.1016/j.devcel.2020.04.005 32369742 PMC7255058

[28] von Gise A Zhou B Honor LB Ma Q Petryk A Pu WT. WT1 regulates epicardial epithelial to mesenchymal transition through *β*-catenin and retinoic acid signaling pathways. Dev Biol. (2011) 356(2):421–31. 10.1016/j.ydbio.2011.05.668 21663736 PMC3147112

[29] Jackson-Weaver O Ungvijanpunya N Yuan Y Qian J Gou Y Wu J PRMT1-p53 Pathway controls epicardial EMT and invasion. Cell Rep. (2020) 31(10):107739. 10.1016/j.celrep.2020.107739 32521264 PMC7425665

[30] von Gise A Pu WT. Endocardial and epicardial epithelial to mesenchymal transitions in heart development and disease. Circ Res. (2012) 110(12):1628–45. 10.1161/CIRCRESAHA.111.259960 22679138 PMC3427736

[31] Streef TJ Smits AM. Epicardial contribution to the developing and injured heart: exploring the cellular composition of the epicardium. Front Cardiovasc Med. (2021) 8:750243. 10.3389/fcvm.2021.750243 34631842 PMC8494983

[32] Trembley MA Velasquez LS de Mesy Bentley KL Small EM. Myocardin-related transcription factors control the motility of epicardium-derived cells and the maturation of coronary vessels. Development. (2015) 142(1):21–30. 10.1242/dev.116418 25516967 PMC4299137

[33] Makino K Long MD Kajihara R Matsueda S Oba T Kanehira K Generation of cDC-like cells from human induced pluripotent stem cells via notch signaling. J Immunother Cancer. (2022) 10(1):e003827. 10.1136/jitc-2021-003827 35101945 PMC8804689

[34] Yu Z Zhou X Liu Z Pastrana-Gomez V Liu Y Guo M KMT2D-NOTCH Mediates coronary abnormalities in hypoplastic left heart syndrome. Circ Res. (2022) 131(3):280–2. 10.1161/CIRCRESAHA.122.320783 35762338 PMC9308708

[35] Ke Y Jian-Yuan H Ping Z Yue W Na X Jian Y The progressive application of single-cell RNA sequencing technology in cardiovascular diseases. Biomed Pharmacother. (2022) 154:113604. 10.1016/j.biopha.2022.113604 36057222

[36] Rossdeutsch A Smart N Dubé KN Turner M Riley PR. Essential role for thymosin *β*4 in regulating vascular smooth muscle cell development and vessel wall stability. Circ Res. (2012) 111(4):e89–102. 10.1161/CIRCRESAHA.111.259846 22723298

[37] Mantri M Scuderi GJ Abedini-Nassab R Wang MFZ McKellar D Shi H Spatiotemporal single-cell RNA sequencing of developing chicken hearts identifies interplay between cellular differentiation and morphogenesis. Nat Commun. (2021) 12(1):1771. 10.1038/s41467-021-21892-z 33741943 PMC7979764

[38] Peng H Xu J Yang XP Dai X Peterson EL Carretero OA Thymosin-*β*4 prevents cardiac rupture and improves cardiac function in mice with myocardial infarction. Am J Physiol Heart Circ Physiol. (2014) 307(5):H741–51. 10.1152/ajpheart.00129.2014 25015963 PMC4187393

[39] Bock-Marquette I Saxena A White MD Dimaio JM Srivastava D. Thymosin beta4 activates integrin-linked kinase and promotes cardiac cell migration, survival and cardiac repair. Nature. (2004) 432(7016):466–72. 10.1038/nature03000 15565145

[40] Qian L Huang Y Spencer CI Foley A Vedantham V Liu L In vivo reprogramming of murine cardiac fibroblasts into induced cardiomyocytes. Nature. (2012) 485(7400):593–8. 10.1038/nature11044 22522929 PMC3369107

[41] Wang YL Yu SN Shen HR Wang HJ Wu XP Wang QL Thymosin *β*4 released from functionalized self-assembling peptide activates epicardium and enhances repair of infarcted myocardium. Theranostics. (2021) 11(9):4262–80. 10.7150/thno.52309 33754060 PMC7977468

[42] Lupu IE Redpath AN Smart N. Spatiotemporal analysis reveals overlap of key proepicardial markers in the developing murine heart. Stem Cell Rep. (2020) 14(5):770–87. 10.1016/j.stemcr.2020.04.002 PMC7221110 32359445

[43] Heinrichs M Ashour D Siegel J Büchner L Wedekind G Heinze KG The healing myocardium mobilizes a distinct B-cell subset through a CXCL13-CXCR5-dependent mechanism. Cardiovasc Res. (2021) 117(13):2664–76. 10.1093/cvr/cvab181 34048536

[44] Lukowski SW Patel J Andersen SB Sim SL Wong HY Tay J Single-Cell transcriptional profiling of aortic endothelium identifies a hierarchy from endovascular progenitors to differentiated cells. Cell Rep. (2019) 27(9):2748–58. e3. 10.1016/j.celrep.2019.04.102 31141696

[45] Andueza A Kumar S Kim J Kang DW Mumme HL Perez JI Endothelial reprogramming by disturbed flow revealed by single-cell RNA and chromatin accessibility study. Cell Rep. (2020) 33(11):108491. 10.1016/j.celrep.2020.108491 33326796 PMC7801938

[46] Werner N Nickenig G Sinning JM. Complex PCI procedures: challenges for the interventional cardiologist. Clin Res Cardiol. (2018) 107(Suppl 2):64–73. 10.1007/s00392-018-1316-1 29978353

[47] Yap C Mieremet A de Vries CJM Micha D de Waard V. Six shades of vascular smooth muscle cells illuminated by KLF4 (krüppel-like factor 4). Arterioscler Thromb Vasc Biol. (2021) 41(11):2693–707. 10.1161/ATVBAHA.121.316600 34470477 PMC8545254

[48] Wirka RC Wagh D Paik DT Pjanic M Nguyen T Miller CL Atheroprotective roles of smooth muscle cell phenotypic modulation and the TCF21 disease gene as revealed by single-cell analysis. Nat Med. (2019) 25(8):1280–9. 10.1038/s41591-019-0512-5 31359001 PMC7274198

[49] Pan H Xue C Auerbach BJ Fan J Bashore AC Cui J Single-Cell genomics reveals a novel cell state during smooth muscle cell phenotypic switching and potential therapeutic targets for atherosclerosis in mouse and human. Circulation. (2020) 142(21):2060–75. 10.1161/CIRCULATIONAHA.120.048378 32962412 PMC8104264

[50] Miano JM Fisher EA Majesky MW. Fate and state of vascular smooth muscle cells in atherosclerosis. Circulation. (2021) 143(21):2110–6. 10.1161/CIRCULATIONAHA.120.049922 34029141 PMC8162373

[51] Gil-Pulido J Amézaga N Jorgacevic I Manthey HD Rösch M Brand T Interleukin-23 receptor expressing *γδ* T cells locally promote early atherosclerotic lesion formation and plaque necrosis in mice. Cardiovasc Res. (2021):cvab359. 10.1093/cvr/cvab359. [Epub ahead of print] 34897380

[52] Zernecke A Winkels H Cochain C Williams JW Wolf D Soehnlein O Meta-Analysis of leukocyte diversity in atherosclerotic mouse aortas. Circ Res. (2020) 127(3):402–26. 10.1161/CIRCRESAHA.120.316903 32673538 PMC7371244

[53] Winkels H Wolf D. Heterogeneity of T cells in atherosclerosis defined by single-cell RNA-Sequencing and cytometry by time of flight. Arterioscler Thromb Vasc Biol. (2021) 41(2):549–63. 10.1161/ATVBAHA.120.312137 33267666 PMC7837690

[54] Vallejo J Cochain C Zernecke A Ley K. Heterogeneity of immune cells in human atherosclerosis revealed by scRNA-Seq. Cardiovasc Res. (2021) 117(13):2537–43. 10.1093/cvr/cvab260 34343272 PMC8921647

[55] Schäfer A König T Bauersachs J Akin M. Novel therapeutic strategies to reduce reperfusion injury after acute myocardial infarction. Curr Probl Cardiol. (2022):101398. 10.1016/j.cpcardiol.2022.101398. [Epub ahead of print] 36108813

[56] Fefer P Knudtson ML Cheema AN Galbraith PD Osherov AB Yalonetsky S Current perspectives on coronary chronic total occlusions: the Canadian multicenter chronic total occlusions registry. J Am Coll Cardiol. (2012) 59(11):991–7. 10.1016/j.jacc.2011.12.007 22402070

[57] Das S Goldstone AB Wang H Farry J D'Amato G Paulsen MJ A unique collateral artery development program promotes neonatal heart regeneration. Cell. (2019) 176(5):1128–42. e18. 10.1016/j.cell.2018.12.023 30686582 PMC6435282

[58] Laugsand LE Strand LB Platou C Vatten LJ Janszky I. Insomnia and the risk of incident heart failure: a population study. Eur Heart J. (2014) 35(21):1382–93. 10.1093/eurheartj/eht019 23462728

[59] Vargas-Valderrama A Ponsen AC Le Gall M Clay D Jacques S Manoliu T Endothelial and hematopoietic hPSCs differentiation via a hematoendothelial progenitor. Stem Cell Res Ther. (2022) 13(1):254. 10.1186/s13287-022-02925-w 35715824 PMC9205076

[60] Ubil E Duan J Pillai IC Rosa-Garrido M Wu Y Bargiacchi F Mesenchymal-endothelial transition contributes to cardiac neovascularization. Nature. (2014) 514(7524):585–90. 10.1038/nature13839 25317562 PMC4214889

[61] Dong W Zhao Y Wen D Lin Y Zeng C Gu J Wnt4 is crucial for cardiac repair by regulating mesenchymal-endothelial transition via the phospho-JNK/JNK. Theranostics. (2022) 12(9):4110–26. 10.7150/thno.71392 35673578 PMC9169355

[62] Wu X Reboll MR Korf-Klingebiel M Wollert KC. Angiogenesis after acute myocardial infarction. Cardiovasc Res. (2021) 117(5):1257–73. 10.1093/cvr/cvaa287 33063086

[63] Dubé KN Thomas TM Munshaw S Rohling M Riley PR Smart N. Recapitulation of developmental mechanisms to revascularize the ischemic heart. JCI Insight. (2017) 2(22):e96800. 10.1172/jci.insight.96800 29202457 PMC5752387

[64] Räsänen M Sultan I Paech J Hemanthakumar KA Yu W He L VEGF-B Promotes endocardium-derived coronary vessel development and cardiac regeneration. Circulation. (2021) 143(1):65–77. 10.1161/CIRCULATIONAHA.120.050635 33203221

[65] Miquerol L Thireau J Bideaux P Sturny R Richard S Kelly RG. Endothelial plasticity drives arterial remodeling within the endocardium after myocardial infarction. Circ Res. (2015) 116(11):1765–71. 10.1161/CIRCRESAHA.116.306476 25834185

[66] Sayed A Turoczi S Soares-da-Silva F Marazzi G Hulot JS Sassoon D Hypoxia promotes a perinatal-like progenitor state in the adult murine epicardium. Sci Rep. (2022) 12(1):9250. 10.1038/s41598-022-13107-2 35661120 PMC9166725

[67] Sanchez-Fernandez C Rodriguez-Outeiriño L Matias-Valiente L Ramirez de Acuña F Hernandez-Torres F Lozano-Velasco E Regulation of epicardial cell fate during cardiac development and disease: an overview. Int J Mol Sci. (2022) 23(6):3220. 10.3390/ijms23063220 35328640 PMC8950551

[68] Zhou B Honor LB He H Ma Q Oh JH Butterfield C Adult mouse epicardium modulates myocardial injury by secreting paracrine factors. J Clin Invest. (2011) 121(5):1894–904. 10.1172/JCI45529 21505261 PMC3083761

[69] Pinto AR Bobik A. Mapping human pluripotent stem cell-endothelial cell differentiation using scRNA-Seq: a step towards therapeutic angiogenesis. Eur Heart J. (2020) 41(9):1037–9. 10.1093/eurheartj/ehz464 31263875

[70] Paik DT Tian L Lee J Sayed N Chen IY Rhee S Large-Scale single-cell RNA-Seq reveals molecular signatures of heterogeneous populations of human induced pluripotent stem cell-derived endothelial cells. Circ Res. (2018) 123(4):443–50. 10.1161/CIRCRESAHA.118.312913 29986945 PMC6202208

[71] McCracken IR Dobie R Bennett M Passi R Beqqali A Henderson NC Mapping the developing human cardiac endothelium at single cell resolution identifies MECOM as a regulator of arteriovenous gene expression. Cardiovasc Res. (2022):cvac023. 10.1093/cvr/cvac023. [Epub ahead of print] PMC9648824 35212715

[72] Alonaizan R Carr C. Cardiac regeneration following myocardial infarction: the need for regeneration and a review of cardiac stromal cell populations used for transplantation. Biochem Soc Trans. (2022) 50(1):269–81. 10.1042/BST20210231 35129611 PMC9042388

[73] Chen P Ning X Li W Pan Y Wang L Li H Fabrication of T*β*4-exosome-releasing artificial stem cells for myocardial infarction therapy by improving coronary collateralization. Bioact Mater. (2022) 14:416–29. 10.1016/j.bioactmat.2022.01.029 35386821 PMC8964820

[74] Tyser RCV Ibarra-Soria X McDole K Arcot Jayaram S Godwin J van den Brand TAH Characterization of a common progenitor pool of the epicardium and myocardium. Science. (2021) 371(6533):eabb2986. 10.1126/science.abb2986 33414188 PMC7615359

[75] Lu J Ahmad R Nguyen T Cifello J Hemani H Li J Heterogeneity and transcriptome changes of human CD8+ T cells across nine decades of life. Nat Commun. (2022) 13(1):5128. 10.1038/s41467-022-32869-x 36050300 PMC9436929

[76] Liu J Ma P Lai L Villanueva A Koenig A Bean GR Transcriptional and immune landscape of cardiac sarcoidosis. Circ Res. (2022) 131(8):654–69. 10.1161/CIRCRESAHA.121.320449 36111531 PMC9514756

